# An Action-Based Fine-Grained Access Control Mechanism for Structured Documents and Its Application

**DOI:** 10.1155/2014/232708

**Published:** 2014-07-17

**Authors:** Mang Su, Fenghua Li, Zhi Tang, Yinyan Yu, Bo Zhou

**Affiliations:** ^1^State Key Laboratory of Integrated Services Network, Xidian University, Xi'an 710071, China; ^2^State Key Laboratory of Information Security, Institute of Information Engineering, Chinese Academy of Sciences, Beijing 100093, China; ^3^Institute of Computer Science & Technology, Peking University, Beijing 100080, China; ^4^Liverpool John Moores University, Liverpool L3 3AF, UK

## Abstract

This paper presents an action-based fine-grained access control mechanism for structured documents. Firstly, we define a describing model for structured documents and analyze the application scenarios. The describing model could support the permission management on chapters, pages, sections, words, and pictures of structured documents. Secondly, based on the action-based access control (ABAC) model, we propose a fine-grained control protocol for structured documents by introducing temporal state and environmental state. The protocol covering different stages from document creation, to permission specification and usage control are given by using the Z-notation. Finally, we give the implementation of our mechanism and make the comparisons between the existing methods and our mechanism. The result shows that our mechanism could provide the better solution of fine-grained access control for structured documents in complicated networks. Moreover, it is more flexible and practical.

## 1. Introduction

The various handheld devices, represented by tablets and smart phones, have brought a novel and collaborative way to access information. It satisfies the increasing requirement for individualized service and makes the information multidimensional in such open networked environment. As a result structured documents, which integrate both content and layout, have become a key information representation in the networked environment of cloud computing, mobile computing, and pervasive computing. While providing the original feeling and the same user experience as paper-based publications, it has speeded up the information transmission significantly. Research on structured documents contains document modeling, analysis, and recognition. At present, the readers and digital contents become more and more various. We expect that the structured document is stable in layout and flexible in content. The content of structured document not only could be texts and pictures, but also integrate both 2D and 3D elements. Moreover, it could interact with the users as well. With the rapid increasing of data represented by structured documents, the granularity of the access control is changing from the whole document to its objects, and the requirement for interaction and permission specification has emerged. However, the current access control model and permission management are mainly realized based on operation system, database, and large-scale information system. The existing access control mechanisms are implemented based on the whole document, so they cannot be adapted to the current situation. Therefore, the description and model of the access control for structured documents have to be changed from the entire document to specific parts of it and from coarse-grained to fine-grained.

Access control is mainly used to solve the problem of data sharing and permission assignment. In the initial stages of access control, there were models such as discretionary access control (DAC) and mandatory access control (MAC). These models have mapped users and permissions directly. Later, role based access control (RBAC) was proposed by introducing the role concept, which has assigned permission to roles instead of users. With the development of the network, the access control models based on temporal state and location have been presented for the purpose of privacy protection. The models could activate and inactivate periodic role or describe how to use the location information in permission determination [1–4]. The wide application of digital communications makes the network become an open, distributed, and complicated system, which supports mobile computing and cloud computing as well. In order to accommodate the distributed environment, UCON and RBAC for multidomain [5, 6] have been proposed. The authors in [7–9] have given the concepts of “attribute” and “action,” respectively, for the future considerations of temporal state and environmental state. At present, the latest challenges for access control are not only the different requirements of security on different devices in various environments, but also the problems of fine-grained management when the users are crossing multiple domains.

The access control for structured document, represented by XML, usually is implemented based on DAC, MAC, and RBAC, without considering temporal state and environmental state. Reference [10] has presented a fine-grained MAC model for XML documents. Its management and control are based on properties of XML. However, because it is based on MAC only, without considerations of temporal and contextual information, it cannot accommodate the demand of structured document access control at anytime and anywhere.

To solve the aforementioned problems, we have done research on structured document description and access control models. In this paper, we present an action-based fine-grained access control mechanism for structured documents by extending the model of [9]. Based on the analysis of application scenarios, we also provide the corresponding security protocols. The protocols take advantage of the current signature encryption technology to ensure the integrity of access control mechanism and provide basis for its implementation. Therefore we can skip the security proof in detail. Finally, we justify our method by comparing it with existing solutions.

The rest of the paper is structured as follows.  Section 2 analyzes the common application scenarios of structured documents. The new access control mechanism of structured documents, including model, protocols, and its implementation, are presented in Section 3. The implementation of our mechanism will give in Sections 4 and 5 which compares the properties of the new mechanism with current ones. And finally we conclude the paper in Section 6.

## 2. Application Scenario Analysis

Users always expect to access any digital contents of structured documents anytime and anywhere. Moreover, they would like to specify the permissions according their own requirement. However, both the creators of the documents and administrators of content servers not only do hope to provide users with convenient and efficient service, but also expect to implement the fine-grained access control of structured documents simultaneously. We analyze three common application scenarios of structured documents in the following subsections.

(1) Document preview: content providers (CP) expect to attract customers to buy the whole digital work by offering a few free contents, while customers often hope to determine the purchase intentions based on their experience of preview.

(2) Individual specification of document permissions: if user B has already purchased the digital contents needed successfully, he could expect to specify permissions and constraints associated with the usage of the contents individually. For instance, he would choose some sections to be a new section and then specify their permission for future usage. Therefore, the permission description of structured documents should be fine-grained and the permissions specification of new document should be flexible.

(3) Multielement restrictions on access to documents: there are three more aspects of the issue. Firstly, the authorization of structured documents usually has a lifetime. After the time, the documents will not be used. Secondly, the contents servers also have restrictions on the number of current online users to ensure the performance. Thirdly, the users' environmental states will play an important role in permission assignment.

## 3. Action-Based Fine-Grained Access Control Mechanism for Structured Documents

Based on the scenario analysis in Section 2, we propose an action-based access control mechanism in this section. In the first subsection, we present a fine-grained description for the structured documents, as our mechanism is built on top of it. In the second subsection, we introduce the action-based access control model for the structured documents based on ABAC. Finally, we describe the corresponding protocols in the last subsection.

### 3.1. A Fine-Grained Description

Structured documents could be described as a 5-tuple* SDoc* = (*S*_*o*_, *o*_*r*_, *S*_*a*_, ∂,* sattr*).

**Figure 1:**
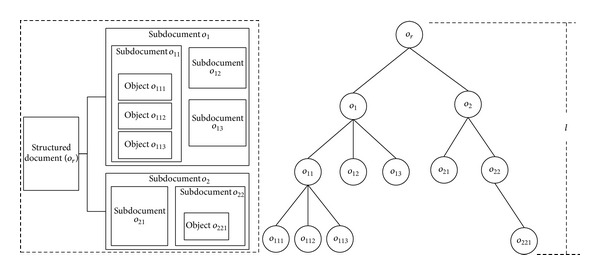
The nesting relationship between objects.

- *S*_*o*_ denotes all the objects sets of structured document* SDoc*. The objects could be anything users want to describe, such as sections, chapters, pages, sentences, and even words.
- *o*_*r*_ denotes the root of objects, where *o*_*r*_ ∈ *S*_*o*_, and it is defined to implement traversal and search for other objects.
- *S*_*a*_ denotes all the objects' attributes sets of structured document. The attributes of objects could be security level, time, location, and so on.
- *sattr* is a binary relationship, where *sattr*⊆*S*_*a*_ × *S*_*o*_. If *a*_1_ ∈ *S*_*a*_, *o*_1_ ∈ *S*_*o*_, and (*a*_1_, *o*_1_) ∈ *sattr*, then *a*_1_ is the security attribute of *o*_1_.
- Function ∂ (∂:*S*_*o*_ → *S*_*o*_) denotes the nesting relationship between objects. If *o*_1_, *o*_2_ ∈ *S*_*o*_ and ∂(*o*_1_) = *o*_2_, then *o*_1_ is nested by *o*_2_. Assuming object *o*_*k*_ is included by document *d* and the object nested by *o*_*k*_ is represented as *o*_*k*−*l*_, *l* is the nesting depth of *o*_*k*_ in *d* when *o*_*k*−*l*_ = *d*. So let ∂_*l*_ = (∂)^*l*^; then ∂(*o*_*k*_) = *o*_*k*−*l*_, (∂(*o*_*k*_))^2^ = ∂(∂(*o*_*k*_)) = *o*_*k*−2_, …, and (∂(*o*_*k*_))^*l*^ = ∂(…∂(*o*_*k*_)) = *o*_*k*−*l*_. The nesting relationship of objects, subdocuments, and documents could be described by multibranches tree. The structured document is the root of the tree, and the nested objects and subdocuments are nodes or leaves. For instance, in Figure 1, (∂(*o*_111_))^*l*^ = *o*_*r*_, *l* = 3. A sensible design of nesting depth could make the fine-grained access control more effective.

### 3.2. Action-Based Access Control Model

**Figure 2:**
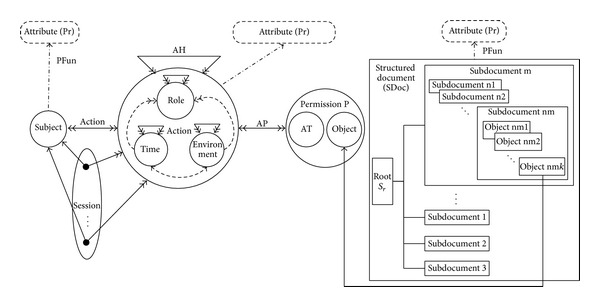
The action-based fine-grained access control model for structured document.

The action-based access control model for structured document is shown in Figure 2. The action hierarchy (AH) and action-permission assignment (AP) are described in [9]. The basic concepts are defined as follows:

- *Subject*: a person or a nature agent that applies to access the resource;
- *Session*: a mapping between a subject and a subset of the roles that the subject is assigned to;
- *Role*: the description of the permission set of subject who wants to access the object at special time and in special place;
- *Time*: the temporal constraint set of subject;
- *Environment: *the environmental constraint set of subject. For instance, location, platform, and other objective conditions related with access control. The system will restrict a subject's permission according to environment when it applies to access;
- *Action*: the set of role, time, and environment of subject when it applies to access;
- *Structured document (SDoc)*: the document is consisted of chapters, sections, fonts, tables, and other elements, including the syntax and semantic of layout;
- *Operation type (AT)*: what a subject could operate on an object. Here ∀*at* ∈ *AT*, and *at* = (*r*, *e*, *a*, *w*). The four symbols mean read, execute, append, and write, respectively. The actual operations such as create, delete, and view could be abstracted to them;
- *Permission (P)*: describes the authorized operation of a subject on a specific object, including the objects and operation type. Here* P* = {*AT*,* Object*};
- *Action *(*Subject* → *Action*): a mapping between subject and action, by which a subject's action information could be obtained.

*Definition*. object is the finest granularity that a subject could access. It is atomic and unified in security requirement. One or more objects could combine a subdocument, and one or more subdocuments could combine a document. We denote the set of objects as* Object*, and in our model it is equal to *S*_*o*_ of the structured document.

In order to solve the fine-grained access control problem of structured document, we extend the model in [9] to 6-tuple (*Subject*, *Action*, *SDoc*, *AT*, *P*, and *Object*). The subject's accessing requirement on objectof structured document* SDoc* for operation type* AT* is denoted as *Req* = (*Subject*, *AT*, *SDoc*, and *Object*).

### 3.3. The Access Control Protocol

The structured documents should be described in sections, pages, or even elements to satisfy the requirement for fine-grained access control. In order to implement our model, we define the corresponding protocols, including document creation, permission specification, and authority management. The symbols details are shown as follows.

*Detailed Descriptions of Symbols*

 Sid: subject's/user's ID,
 Oid: object's ID,
 
*t*_*s*_: subject's temporal state,
 
*e*_*s*_: subject's environmental state,
 
*T*_*s*_: starting time of policy,
 
*T*_*e*_: end time of policy,
 
*k*_*o*_: the object's key symmetry encryption, it is generally encrypted under the subject's private key,
 at: operation type,
 pd: the policy's description, it could be described in XML. Our paper will not discuss it in detail due to the paper length,
 Sk_*A*_: A's private key.

The* NAME* denotes the abstract data types, including the action, role, time, environment, and structured document. The* ROLES* denotes the set of roles.* TSTATES* denotes the set of temporal states.* ESTATE *denotes the set of environmental states.* OBJECTS* denotes the set of objects.* POLICYS* denotes the set of policies. We give the functions for protocol in Z-notation as follows.

- GenSDoc: content provider generates the structured document* SDoc* in specified format (format) by document-packaged tools.
   
*GenSDoc (contents: NAME; out SDoc: NAME) *⊲
   
*SDoc = format (contents); *⊳
- Define Object: user divides the* SDoc* into sections according to their requirement for access control.
   
*Define Object (SDoc: NAME; out OBJECTS: NAME) *⊲
   
*if object *∉* OBJECTS then OBJECTS' = OBJECTS *∪ { *object*}⊳
- Assign Policy: user assigns the policy to the divided SDoc and submits the result to the policy server, where Policy = {*P*_1_, *P*_2_,…, *P*_*k*_}.
   
*Assign Policy (Object, environment, temporal, at, key, Ts, Te, pd: NAME; out Policy: NAME) *⊲
   
*if policy *∉* Policy*
   
*then policy.role = role*
   
*policy.temporal = temporal*
   
*policy.environment = environment*
   
*policy.object = Object*
   
*policy.at = at*
   
*policy.k = key*
   
*policy.Ts = Ts*
   
*policy.Te = Te*
   
*policy.pd = pd*
   
*POLICYS' = POLICYS *∪ { *policy* }
   
*POLICYS= POLICYS' *⊳
- Verifyid: verify the user's identity and certificate; if legal, then return “True,” otherwise return “False.”
   
*Verifyid (user, certification: NAME; out result: BOOLEAN) *
   ⊲*result = (user *∈* U) (isvalid (certification)) *⊳
- GetReq: obtain the user's requirement* req.*
   
*GetReq (user, object, SDoc, at:NAME; out req: NAME) *⊲
   
*req.subject = user*
   
*req.at = at*
   
*req.Object = object*⊳
- GetAction: obtain the information including role* R*, environment* E*, and time* T* for the policy server's further judgment. The information received is (*Role, E, T, and Object*).
   
*GetAction (req, temporal, environment: NAME; out action: NAME) *⊲
   
*action.role = req.subject*
   
*action.temporal = temporal*
   
*action.environment = environment*⊳
- Verifyt: when the user's requirement* req* and action information* action* are obtained, the policy server will validate the user's temporal information by this function. If valid, then return “True,” otherwise return “False.”
   
*Verifyt *(*action, validt: NAME; out result: BOOLEAN*)⊲*action.temporal *∈* TSTATES; validt *⊆* TSTATES*
   
*result = *(∃*t*_1_, *t*_2_∈* validt*·*t*_1_≤* action.temporal *≤*t*_2_)⊳
- Verifye: when the user's requirement* req* and action information* action*are obtained, the policy server will validate the user's environmental information by this function. If valid, then return “True,” otherwise return “False.”
   
*Verifye *(*action, valide: NAME; out result: BOOLEAN*)⊲
   
*action.environment *∈* ESTATES; valide *⊆* ESTATES*
   
*result = *(∃*e*_1_, *e*_2_∈* valide*·*e*_1_≤* action.environment *≤*e*_2_)⊳
- Verifyr: when the user's requirement* req* and action information* action*are obtained, the policy server will validate the user's role by this function. If valid, then return “True,” otherwise return “False.”
   
*Verifyr *(*action, validr: NAME; out result: BOOLEAN*)⊲
   
*action.role *∈* ROLES; validr *⊆* ROLES*
   
*result = *(∃*r*_1_, *r*_2_∈* validr r*_1_≤* action.role *≤*r*_2_)⊳
- JusUsage: the policy server will search the corresponding policy for user according to his action and requirement.
   
*JusUsage (action, req: NAME; out poicy: NAME*)⊲
   
*policy' = *(∃* policy' *∈* POLICY *(*policy'.object = req.object *∧* policy'.at = req.at*))
   
*if verifyr *(*action, policy'.role*)* then*
   
*if verify *(*action, policy'.temporal*)* then*
   
*if verifye *(*action, policy'.environment*)* then*
   
*policy = policy' *⊳

**Figure 3:**
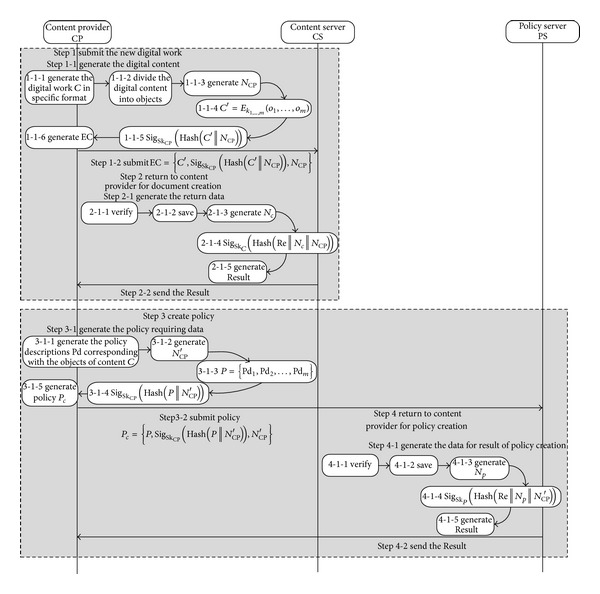
The process of structured documents creation.

*(1) The Protocol for Structured Documents Creation.* By using corresponding tools, the owner of the resource will package his text, video, audio, picture, and 3D objects into one digital work and assign policies according to their own requirement (see Figure 3). The steps of the protocol are explained as follows.

**Step 1 .**

CP→CS the content provider CP creates the digital work and submits it to the content server.

 Step 1-1 Generate the digital content EC.
   Step 1-1-1 Call the function GenSDoc() to generate the digital content* C* in corresponding format.
   Step 1-1-2 Call the function DefineObject() to divide the digital content into objects {*o*_1_,…, *o*_*m*_} according to the requirement.
   Step 1-1-3 Generate the random number *N*_CP_.
   Step 1-1-4 Encrypt the objects *o*_1_,…, *o*_*m*_ obtained from Step1-1-2 under the session key *k*_1_,…, *k*_*m*_ exchanged before. The cipher text is *C*′ = *E*_*k*_1_,…,*k*_*m*__(*o*_1_,…, *o*_*m*_).
   Step 1-1-5 Generate the signature Sig_Sk_CP__(Hash(*C*′||*N*_CP_)).
   Step 1-1-6 Generate the final digital content EC = {*C*′, Sig_Sk_CP__(Hash(*C*′||*N*_CP_)), *N*_CP_}.
 Step 1-2 Submit the digital content EC to the content server CS.

**Step 2 .**

CS→CP content server returns the result of new content submission to content provider.

 Step 2-1 Generate the data for result.
   Step 2-1-1 Verify the information by using the CP's public key. If failed, go to Step 5.
   Step 2-1-2 Save the digital content EC, and add the new content information to the publishing list. Generate the basic data Re for result.
   Step 2-1-3 Generate random number *N*_*c*_.
   Step 2-1-4 Generate the signature Sig_Sk_*C*__(Hash(*Re*||*N*_*C*_||*N*_CP_)).
   Step 2-1-5 Generate the final data for returning result* Result = *(Re, Sig_Sk_*C*__(Hash(*Re*||*N*_*C*_||*N*_CP_)), *N*_C_, *N*_CP_).
 Step 2-2 Send* Result* to content provider CP.

**Step 3 .**

CP→PS the content provider CP creates the policies and submits it to the policy server.

 Step 3-1 Generate the policy requiring data.
   Step 3-1-1 Generate the policy descriptions Pd_1_, Pd_2_,…, Pd_*m*_ corresponding with the objects of content* C*.
   Step 3-1-2 Generate the random number *N*_CP_′.
   Step 3-1-3 Policy *P* = {Pd_1_, Pd_2_,…, Pd_*m*_}.
   Step 3-1-4 Generate the signature Sig_Sk_CP__(Hash(*P*||*N*_CP_′)).
   Step 3-1-5 Generate the user's policy *P*_*c*_ = {*P*, Sig_Sk_CP__(Hash(*P*||*N*_CP_′)), *N*_CP_′}.
 Step 3-2 Submit the *P*_*c*_ to policy server.

**Step 4 .**

PS→CP policy server returns result to content provider for policy creation.

 Step 4-1 Generate the data for result of policy creation.
   Step 4-1-1 Verify the information by using the CP's public key. If failed, go to Step 5.
   Step 4-1-2 Save the policy, and generate the basic data Re for result.
   Step 4-1-3 Generate the random number *N*_*p*_.
   Step 4-1-4 Generate the signature Sig_Sk_*P*__(Hash(*Re*||*N*_*p*_||*N*_CP_′)).
   Step 4-1-5 Generate the final data for result* Result *= (re, Sig_Sk_*P*__(Hash(*Re*||*N*_*p*_||*N*_CP_′)), *N*_*p*_, *N*_CP_′).
 Step 4-2 Send* Result *to content provider CP.

**Step 5 .**

End of Creation.

**Figure 4:**
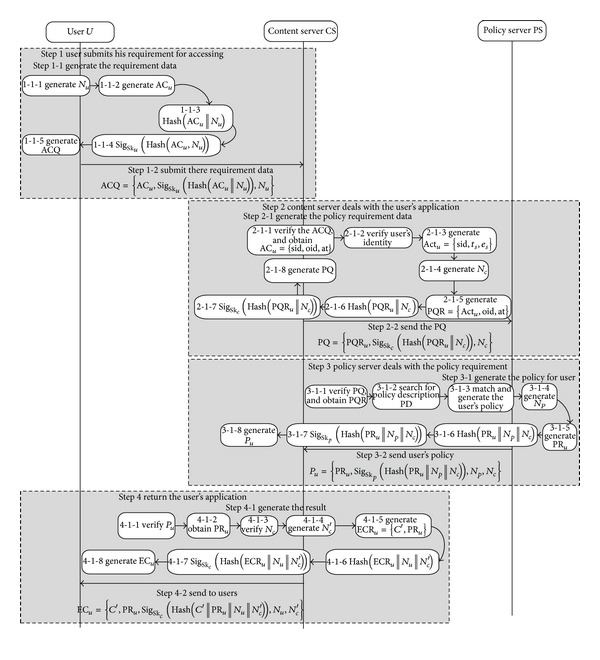
The process of users' authorization.

*(2) The Protocol of Authorization.* User *U* registers the information to the system and obtains a role that can access the system. When *U* needs to access the resource, content server and policy server will return the corresponding digital content and policy according to their role, environment, and temporal states (see Figure 4). The steps of the protocol are explained below.

**Step 1 .**

U→CS user *U* submits the requirement for accessing a digital content to content server according to the resource list of the system.

 Step 1-1 Generate the requirement data.
   Step 1-1-1 Generate the random number *N*_*u*_.
   Step 1-2-1 Generate the requirement basic data AC⁡_*u*_ = {sid, oid, op}.
   Step 1-3-1 Generate Hash(sid | |oid | |op | |*N*_*u*_).
   Step 1-4-1 Generate the signature Sig_Sk_*u*__(Hash(sid||oid||op||*N*_*u*_)).
   Step 1-5-1 Generate the ACQ = {AC_*u*_, Sig_Sk_*u*__(Hash(sid||oid||op||*N*_*u*_)), *N*_*u*_}.
 Step 1-2 Submit the* ACQ *to content server.

**Step 2 .**

CS→PS content server in data center deals with the user's requirement.

 Step 2-1 Generate the policy requirement data.
   Step 2-1-1 Analyze the user's requirement data* ACQ*, and verify the data by using the user's public key. If failed, go to Step 5.
   Step 2-1-2 Call function GetReq() to obtain the user's requirement* req*, and verify the legitimacy of user's identity; if failed, go to Step 5.
   Step 2-1-3 Call function GetAction() to generate the user's action information Act_*u*_ = {sid, *t*_*s*_, *e*_*s*_}.
   Step 2-1-4 Generate the random number *N*_*c*_.
   Step 2-1-5 Generate the user's policy requirement basic data PQR = {Act_*u*_, oid, op}.
   Step 2-1-6 Generate PQ_Hash = Hash(sid||*t*_*s*_||*e*_*s*_||oid||at||*N*_*C*_).
   Step 2-1-7 Generate signature Sig_Sk_*c*__(PQ_Hash).
   Step 2-1-8 Generate the policy requirement data PQ = {PQR_*u*_, Sig_Sk_*c*__(PQ_Hash), *N*_*C*_}.
 Step 2-2 Send PQ to the policy server.

**Step 3 .**

PS→CS the policy server in data center deals with the user's policy requirement.

 Step 3-1 Generate the policy for user.
   Step 3-1-1 Analyze the policy requirement data PQR, and verify the data by using the CP's public key. If failed go to Step 5.
   Step 3-1-2 Obtain the information sid, oid, at,* t*_*s*_,* e*_*s*_ from the requirement, and search in the policy database for the corresponding policy description PD according to the information.
   Step 3-1-3 Call function* JusUsage*() to match the user's requirement and generate the user's policy.
   Step 3-1-4 Generate random number *N*_*p*_.
   Step 3-1-5 Generate the user's policy basic data PR_*u*_ = {sid, oid, at, *T*_*s*_, *T*_*e*_, *k*_*o*_, Pd}.
   Step 3-1-6 Generate PR_Hash = Hash(PR_*u*_||*N*_*P*_||*N*_*C*_).
   Step 3-1-7 Generate signature Sig_Sk_*p*__(PR_Hash).
   Step 3-1-8 Generate the policy data *P*_*u*_ = {PR_*u*_, Sig_Sk_*p*__(PR_Hash), *N*_*p*_, *N*_*C*_}.
 Step 3-2 Send *P*_*u*_ to content server.

**Step 4 .**

CS→U data center returns the result to user.

 Step 4-1 Generate the result for return
   Step 4-1-1 Analyze the policy data *P*_*u*_, and verify the data by using the PS's public key. If failed, go to Step 5.
   Step 4-1-2 Obtain the policy basic data PR_*u*_.
   Step 4-1-3 Verify the random number *N*_*c*_. If failed, go to Step 5.
   Step 4-1-4 Generate the random data *N*_*c*_′.
   Step 4-1-5 Generate the basic data ECR = {*C*′, PR_*u*_} for return.
   Step 4-1-6 Generate ECR_Hash = Hash(*C*′||PR_*u*_||*N*_*c*_′).
   Step 4-1-7 Generate signature Sig_Sk_*c*__(ECR_Hash).
   Step 4-1-8 Generate EC_*u*_ = {ECR, Sig_Sk_*C*__(ECR_Hash)), *N*_*c*_′, *N*_*u*_}.
 Step 4-2 Return the EC_*u*_ to the user.

**Step 5 .**

End of authorization.

*(3) The Protocol for User to Read Structured Document.* Generally, the user could read the digital content by using the specific readers. When the user obtains the final return data* ECR*, his reader will verify the signature and random number. If succeeded, it will analyze the data in three steps. Firstly, the reader gets the *T*_*s*_ and *T*_*e*_ from* PR*_*u*_ and verifies this policy. Secondly, it obtains the session key *k* and cipher text *C*′. Policy server could encrypt the key *k* with the user's public key, and the reader can decrypt it with the user's private key. Finally, the reader will analyze the permission description* Pd* and compute *D*_*k*′_(*C*′) to obtain the corresponding permission. After obtaining the permission description, the reader will present the corresponding digital content to the user.

*(4) Security Analysis.* Because the digital content has been encrypted before submission, our protocol ensures the confidentiality of the data. The requirement and data packages transferred among the users, content providers, content servers, and policy servers have been signed. Therefore the integrity and nonrepudiation can be ensured. In addition, the random numbers have been used during the transmission to withstand the replaying attack and man-in-the-middle attack.

## 4. Implementation of Our Mechanism

**Figure 5:**
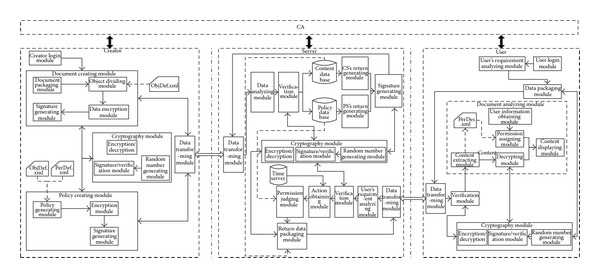
Framework for implementation.

Our system is under Windows based on C/S model, and the framework for implementation is shown in Figure 5. The server side consisted of the content server and policy server and the client side includes user interface and creator interface. The server side consists of user's requirement analysis module, verification module, action obtaining module, permission assignment module, and the other universal modules like cryptography and data transforming modules. The user's requirement analysis module could analyze the data packages form the users and divide them into the data stream for document creating, policy creating, or data accessing. The verification module will verify the integrity and the random number by calling the cryptography module. The action obtaining module will get the user's role, temporal state, and environmental state together to generate the action. The user's role is obtained according to* sid*. The temporal state from the time server and the environmental state includes the network, physical location, platform, or the software. In our system, we take the network and software for experiment, and we will improve the other factors in the future. The network information is obtained from the user's data packages and the software information is initialized at the beginning. The client will send the Hash of the client software to server when accessing the resource. The permission assignment module makes the decision according to the policy data base. The format of this data base is (sid, oid, at,* t*_*s*_,* e*_*s*_,* perDes.xml*). Besides, the common modules like data based managing and storing will not be described here.

The client side includes two types, creator and user. The creator mode includes the document packaging module, object dividing module, encryption module, signature generating module, policy generating module, and some general modules for cryptography and data transforming. Document packaging module will compress and package the resource uploaded by the creator and generate the structured file in certain format. The object dividing and encrypting module will define the objects and encrypt them according to the file objDef.xml. The policy generating module will return the permission describing file perDes.xml based on the files objDef.xml and perDef.xml. The user mode consists of user's requirement analyzing module, document analyzing module, user login module, and some general modules for cryptography and data transforming. The user login module will get the username and password. And, then, it will decide the user's role information and pass it the system. The user's requirement analyzing module will analyze the user's requirement and abstract the sid, oid, at to the requirement basic data for system. Document analyzing module includes the submodules like content extracting, decrypting, user information obtaining, and permission assigning. Firstly, this module extracts the content and policy and then gets the user's corresponding information, for example, role and time network address, according to the policy description. Finally, it will display the right data and permission for user.

The XML files mentioned above will be introduced as follows and the analysis of them is implemented based on the ParseXML.

(1)* objDef.xml* describes the division of objects, including the object name, object ID, parent object, and content. For example, an object named “Introduction,” whose ID is “O11” and parent object is “O1”, could be described in Algorithm 1. 

(2)* perDef.xml* describes the permission definition of the certain object, including the object ID, operation type, and action description. For example, the situation that role A can read and write the object O11 with the IP from 172.16.1.5 to 172.16.1.35 between 8:00 am and 10:00 am could be described in Algorithm 2.

(3)* perDes.xml *describes the permission information for analysis of client, including the sid, oid, the begin and end time, key information, and the description of permission. According to (1) and (2), the* perDes.xml* for “User_A” to access “O11” is as Algorithm 3.

The tag<ko> is defined to describe the key information. This key is encrypted by the key embedded in the client and the user cannot obtain it, so the user can obtain the right data only by the certain client.

By testing, the system has realized the functions of data encryption, data decryption, and access control according to the temporal state and network address. Also, the users can only use the data on the certain client equipment. Because the client will do the integrity authentication by communicating with the server, the user cannot access by tampering the client and cannot decrypt the data without the client. The keys of our system are managed by the certificate authentication (CA) in Figure 5. The CA realizes the functions of certificate creating, distributing, and managing. The symmetric keys will be generated by the creating clients and managed by the servers. Our main focus, however, is on the designing and implementation of the access control mechanism for structured document based on ABAC (action-based access control); the new technology to manage the keys, such as key derivation functions or key hierarchy, will be discussed in the future.

**Figure 6:**
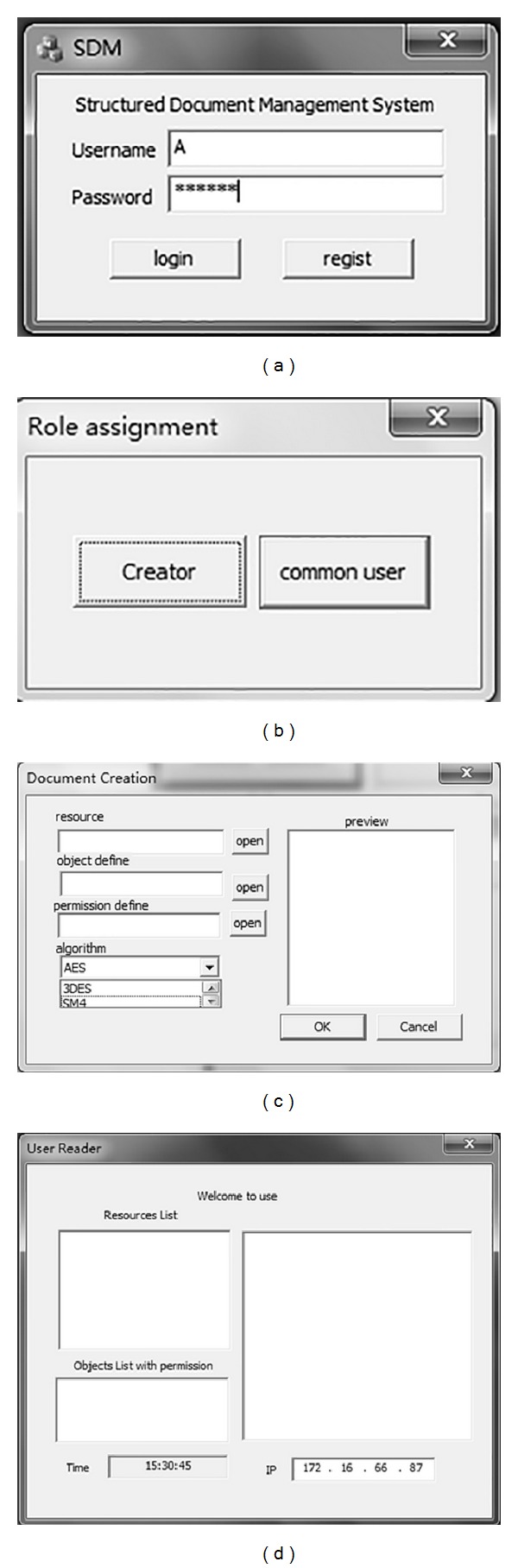
The user interface of the system.

The implemented results of the system are shown in Figure 6. The user should login the system (see Figure 6(a)) and choose the role (see Figure 6(b)). After login and role assignment, the user can create the resource by “Document Creation” (see Figure 6(c)) or access the resource by “User Reader” (see Figure 6(d)). When creating a new resource, the creator can choose the resources for packaged, decide the algorithm for encryption, and upload the XML files for definitions of object and permission.

**Figure 7:**
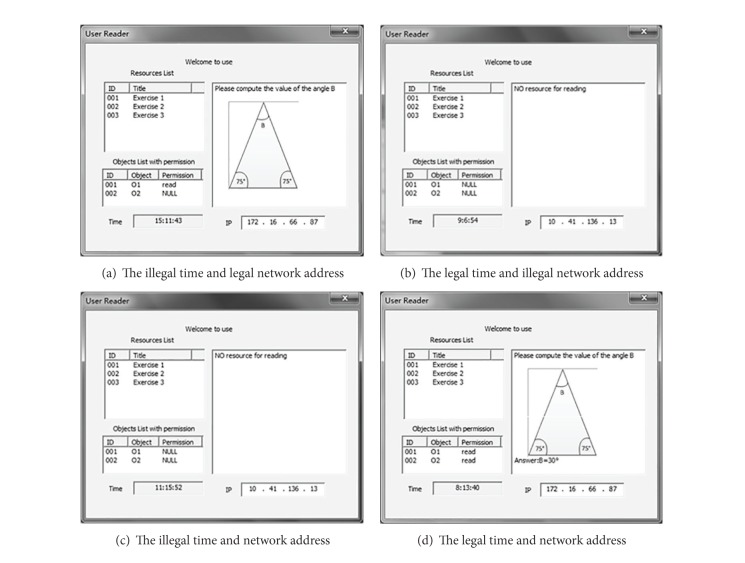
The results of experiments.

Let us make an assumption that a creator has created the resource called “Exercise 1” including the object O1 and object O2. O1 is the questions parts and O2 is the answers parts. User A could apply for permission to access in the classroom (network address: 172.16.66.5–172.16.66.90) and they can read O1 and O2 between 8:00 am and 10:00 am; otherwise, he can only read O1. If the user is out of the classroom, the accessing requirements are always illegal. Results of some experiments are shown in Figure 7. The four situations of the illegal time and legal network address, the legal time and illegal network address, the illegal time and network address, and the legal time and network address are shown in Figures 7(a), 7(b), 7(c), and 7(d), respectively.

## 5. The Properties of Action-Based Access Control for Structured Documents

In this section, we will make the comparison between the current models and ours. The result is shown in Table 1. GB-RBAC [11] and H-RBAC [12] give the group-based and attribute-based access control for distributed computing and cloud computing, respectively. Nevertheless, those models are insufficient for the complicated network without temporal and environmental constraints. GTRBAC [1] extends the temporal constraint of users and allows expressing periodic as well as duration constraints on roles, user-role assignments, and role-permission assignments. LRBAC (location-aware RBAC) [2] has extended RBAC to incorporate the notion of location, but the temporal constraint was ignored. TLRBAC [3] and RBAC model based on space, time, and scale have introduced the temporal state and simple environmental state, but the environmental state only can describe the single physical location which is not enough. In order to describe the permission in more details, paper [7] presents the attribute-based access control and its formal definition. It abstracts temporal state and environmental state as attributes. The permission assignment is based on attributes. However, the description of attributes is too complex, and the concept role is weakened. Action-based access control [9] integrates the temporal state, environmental state, and role and discusses the description of environment in detail. Therefore, it could have wide application in the MLS systems. In the complicated network environment, flexibility and extensibility have become the vital requirements of research on access control. Paper [13] proposes a trust and role based access control framework in infrastructure-centric environment. The scheme is flexible and scalable. Paper [14] presents a dynamic access control model based on description logic (DL). It could assign permission to a user according to his role in a given context. Moreover, composed context is supported. However, all the models mentioned above have not paid enough attention to the fine-grained access control, so they could not accommodate the requirement for structured document. According to the description of XML, paper [10] gives a fine-grained mandatory access control model and its rules for XML documents. Nevertheless, the model's only authority is based on the users' security level and that of XML elements or attributes. Thus it is not suitable for the multielement access control in the current complicate network.

We propose an action-based fine-grained access control for structured document by analyzing the advantages and disadvantages of the models and mechanisms above. Our mechanism is extended from ABAC [9]. It could implement fine-grained description of the structured document and the authority is based on action in the complicated network.

## 6. Conclusions

To solve the problem of fine-grained access control for structured documents in the current complicated network, this paper proposes an action-based access control mechanism. By defining the objective describing model, it could support the permission management on chapters, pages, sections, words, and pictures of structured documents. The corresponding protocols are given to cover different stages in the lifecycle of structured document, including document creation, users' authorization, and user's usage. Other requirements such as preview, combination, and permission specification are accommodated as well. Meanwhile, the confidentiality, integrity, and nonrepudiation are ensured by our mechanism too. In the future research, we intend to implement the access control mechanism with considerations of more factors and address the problem of dynamic adjustment and combination for policy.

## Figures and Tables

**Algorithm 1:**
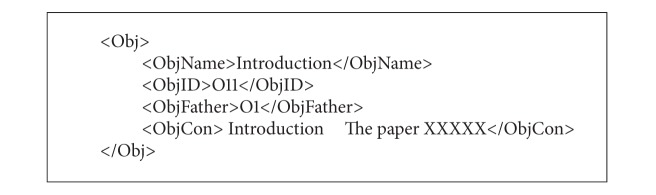


**Algorithm 2:**
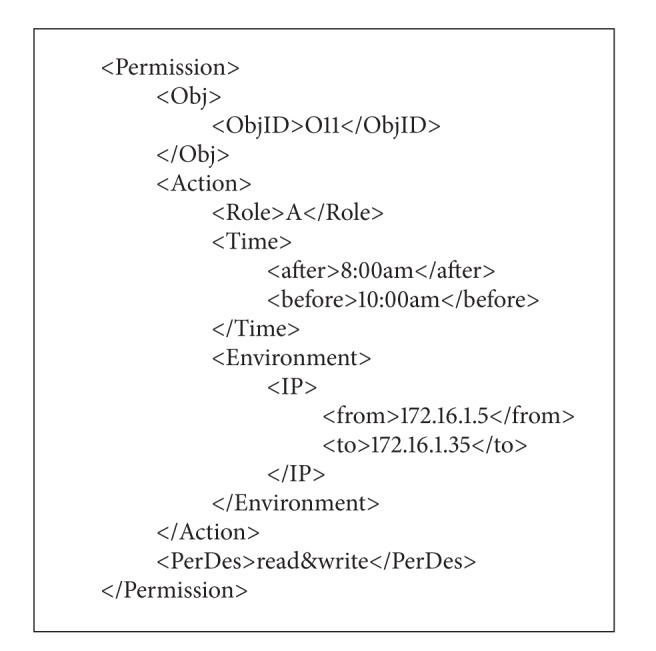


**Algorithm 3:**
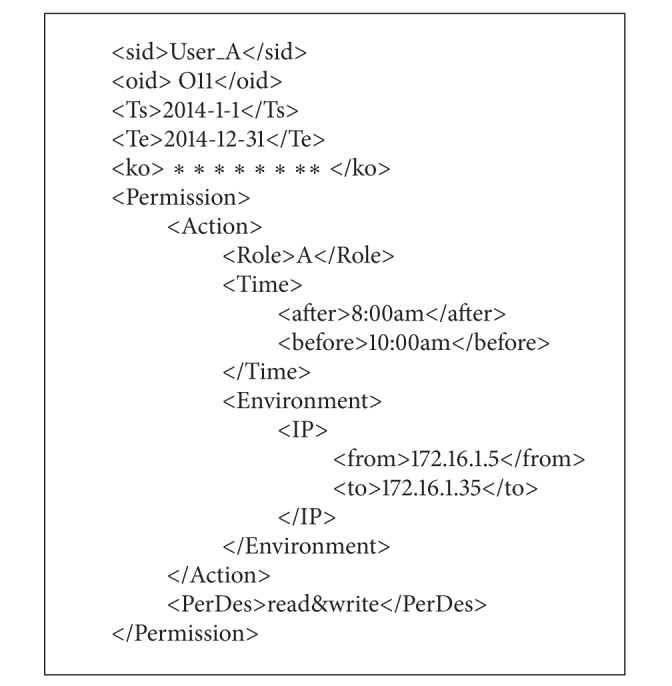


**Table 1:** Comparison between our model and current models.

	Property				
Model	Role	Temporal state	Environmental state	Fine-grained	Permission specification
GB-RBAC [11]	YES	NO	NO	NO	NO
H-RBAC [12]	YES	NO	NO	NO	NO
GTRBAC [1]	YES	YES	NO	NO	NO
LRBAC [2]	YES	NO	PART	NO	NO
TLRBAC [3]	YES	YES	PART	NO	NO
Reference [4]	YES	YES	PART	NO	NO
Attribute-based access control [7]	NO	YES	YES	NO	NO
Action-based access control [9]	YES	YES	YES	NO	NO
ITRBAC [13]	YES	NO	PART	NO	NO
Reference [14]	YES	NO	PART	NO	NO
Reference [10]	YES	NO	NO	PART	NO
Our model	YES	YES	YES	YES	YES
